# Natural Compound Resveratrol Attenuates TNF-Alpha-Induced Vascular Dysfunction in Mice and Human Endothelial Cells: The Involvement of the NF-κB Signaling Pathway

**DOI:** 10.3390/ijms222212486

**Published:** 2021-11-19

**Authors:** Palanisamy Nallasamy, Zi Yae Kang, Xiaolun Sun, Pon Velayutham Anandh Babu, Dongmin Liu, Zhenquan Jia

**Affiliations:** 1 Department of Biology, University of North Carolina at Greensboro, Greensboro, NC 27412, USA; samy.nallasamy@unmc.edu (P.N.); z_kang@uncg.edu (Z.Y.K.); 2 Cell and Molecular Biology (CEMB), University of Arkansas, Fayetteville, AR 72701, USA; xiaoluns@uark.edu; 3 Center of Excellence for Poultry Science, University of Arkansas, 1260 W Maple Street O-409, Fayetteville, AR 72701, USA; 4 Department of Nutrition and Integrative Physiology, College of Health, University of Utah, Salt Lake City, UT 84112, USA; anandh.velayutham@utah.edu; 5 Departments of Human Nutrition, Foods, and Exercise, College of Agriculture and Life Sciences, Virginia Tech, Blacksburg, VA 24061, USA; doliu@vt.edu

**Keywords:** resveratrol, physiological concentrations, vascular inflammation, monocyte adhesion, TNF-α, NF-κB

## Abstract

Resveratrol, a natural compound in grapes and red wine, has drawn attention due to potential cardiovascular-related health benefits. However, its effect on vascular inflammation at physiologically achievable concentrations is largely unknown. In this study, resveratrol in concentrations as low as 1 μm suppressed TNF-α-induced monocyte adhesion to human EA.hy926 endothelial cells (ECs), a key event in the initiation and development of atherosclerosis. Low concentrations of resveratrol (0.25–2 μm) also significantly attenuated TNF-α-stimulated mRNA expressions of MCP-1/CCL2 and ICAM-1, which are vital mediators of EC-monocyte adhesion molecules and cytokines for cardiovascular plaque formation. Additionally, resveratrol diminished TNF-α-induced IκB-α degradation and subsequent nuclear translocation of NF-κB p65 in ECs. In the animal study, resveratrol supplementation in diet significantly diminished TNF-α-induced increases in circulating levels of adhesion molecules and cytokines, monocyte adhesion to mouse aortic ECs, F4/80-positive macrophages and VCAM-1 expression in mice aortas and restored the disruption in aortic elastin fiber caused by TNF-α treatment. The animal study also confirmed that resveratrol blocks the activation of NF-κB In Vivo. In conclusion, resveratrol at physiologically achievable concentrations displayed protective effects against TNF-α-induced vascular endothelial inflammation in vitro and In Vivo. The ability of resveratrol in reducing inflammation may be associated with its role as a down-regulator of the NF-κB pathway.

## 1. Introduction

Cardiovascular disease (CVD) is the number one cause of death in the United States and one of the top leading causes of death worldwide, mostly due to the westernization of traditional diets [1,2,3]. Atherosclerosis, a major cause of CVDs, is an inflammatory vessel disorder commonly characterized by plaque formation as a result of monocyte-derived macrophages that ultimately develop into lipid-laden foam cells [4,5,6]. Previous studies have reported that endothelial dysfunction following chronic inflammation is essential in the initiation and development of atherosclerosis [7,8,9]. In the early stages of atherosclerotic plaque development, circulating monocytes are recruited by activated endothelial cells (ECs) followed by EC-monocyte adhesion and subsequent transmigration into the intima [5]. Accumulating evidence suggests that these processes are driven by proinflammatory chemokines, such as interleukin-8 (IL-8) and monocyte chemoattractant protein-1 (MCP-1), and adhesion molecules, such as intracellular adhesion molecule-1 (ICAM-1) and vascular cell adhesion molecule-1 (VCAM-1) [6,10,11].

It is well-established that tumor necrosis factor-alpha (TNF-α), a major pleiotropic proinflammatory cytokine, plays a pivotal role in endothelial dysfunction and subsequent damage to vascular function [12,13]. Indeed, elevated levels of circulating TNF-α were found in the plasma of humans with vascular diseases [14,15], while in TNF-α knockout mouse models, decreased endothelial adhesion and atherogenesis have been reported [16]. TNF-α is also known to induce apoptosis in aortic endothelial cells [17] and demonstrate a high presence in atherosclerotic lesions [18], indicating its critical role in developing vascular disease. In research, TNF-α has been commonly used as an inflammation trigger due to its ability to increase expression of other proinflammatory cytokines, chemokines, such as MCP-1, and adhesion molecules, including VCAM-1 and ICAM-1 [19,20]. Previous studies reported that TNF-α-induced up-regulation of chemokine and adhesion molecule gene expression is mediated largely by nuclear factor-kappa (NF-κB) [21,22]. NF-κB can be activated upon phosphorylation of inhibitors of NF-κB by TNF-α-stimulated activation of the IkB kinase (IKK) complex [23]. The p65 heterodimer, also known as RelA, is a member of the NF-κB family of transcription factors and shows increased nuclear translocation in the thickened intima of human atherosclerotic lesions [24,25]. Since inflammation-driven endothelial dysfunction is a prime trigger in atherosclerosis initiation and exacerbation, compounds that attenuate TNF-α- induced NF-κB activation and subsequent expression of inflammatory markers are potential therapies to vascular endothelial dysfunction.

Resveratrol (3,5,4′-trihydroxy-trans-stilbene) is a stilbenoid phytoalexin compound found in skins of grapes and berries and naturally present in high concentrations in red wine [26]. Resveratrol has drawn wide attention due to potential cardiovascular-related health benefits potentially stemming from its role as an antioxidant and anti-inflammatory agent [27,28,29,30]. Indeed, results from a study done in a cell-free system indicated resveratrol as a scavenger for superoxide anion radicals [29]. Additionally, data from in vitro studies suggest resveratrol inhibits LPS-induced ROS generation and Nox1 expression, protecting vasculature by reducing oxidative stress [30]. Animal studies demonstrated that resveratrol treatment decreased neutrophil infiltration into myocardial ischemia/reperfusion tissue [27] and reduced cardiac hypertrophy [28], indicating a cardioprotective role. While these data shed light on protective effects of resveratrol against vascular disease, they do not reflect physiological effects of resveratrol as the concentrations used in these studies exceed plasma resveratrol levels (≤5 μm) that are attainable in animals and humans after consumption of resveratrol-containing food or supplements [31,32,33]. In a study done with 12 healthy males aged 25–45 years, depending on whether 25 mg resveratrol was delivered by vegetable juice, wine or grape juice, the peak serum concentration of free and conjugated resveratrol was 1.8–2 μm [31,32]. In another phase I study, up to 2.4 μm of unmetabolized resveratrol was found in the plasma of human participants who orally ingested a single dose of 5 g of resveratrol [33]. When 5 g of resveratrol was ingested daily for 29 consecutive days, peak plasma concentrations of trans-resveratrol reached up to 4.2 μm [34]. In both studies, oral intake of high doses (5 g) of resveratrol was demonstrated to be safe, as evidenced by the lack of any serious adverse events [33,34]. Since most of the previous studies used resveratrol concentrations well above those that were nutritionally relevant, the biological significance of previous findings is largely unclear, and the cellular and organismal action of resveratrol at physiologically achievable concentrations in the plasma (≤5 μm) needs to be examined further. In this study, we investigated whether resveratrol at physiologically achievable concentrations attenuates TNF-α-induced adhesion of monocytes to endothelial cells and its underlying mechanisms. We also analyzed the effect of dietary intake of resveratrol on TNF-α-induced vascular inflammation in C57BL/6 mice.

## 2. Results

### 2.1. Resveratrol Reduced TNF-α-Induced Monocyte Adhesion to ECs

**Figure 1:**
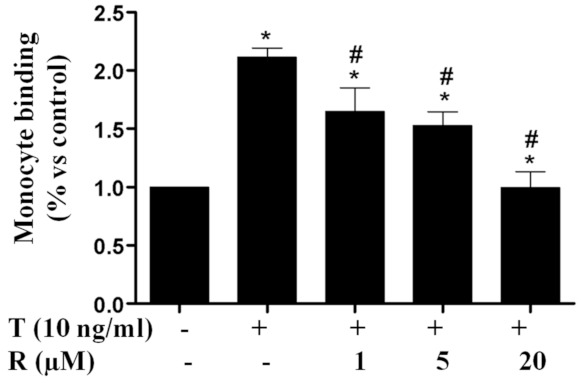
Resveratrol suppressed TNF-α-stimulated monocyte adhesion to EA.hy926 endothelial cells. The cells were pretreated with resveratrol (R, 1 µM, 5 µM, 20 µM) for 1 h prior to the addition of TNF-α (T, 10 ng/mL) for 24 h in the continued presence or absence of resveratrol. THP-1 cells were labeled with a fluorescence probe and the adhesion was determined using a microplate reader at excitation and emission wavelengths of 496 nm and 520 nm. T, TNF-α; R, Resveratrol. Values represent mean ± SEM, *n* = 3–5. *, *p* < 0.05 vs. control; #, *p* < 0.05 vs. TNF-α-alone-treated cells.

Adhesion of monocytes to ECs is a crucial step in propagating endothelial dysfunction in inflammatory diseases. We investigated whether resveratrol would have an anti-inflammatory effect by affecting monocyte adhesion to ECs. Exposure of EA.hy926 ECs to TNF-α showed at least a twofold increase in THP-1 monocyte adhesion to ECs (Figure 1). However, 1 h pretreatment with resveratrol in concentrations as low as 1 μm significantly suppressed TNF-α-induced monocyte binding, and 20 μm resveratrol reduced monocyte adhesion to levels seen in the control group that was not treated with TNF-α. The inhibitory effect of resveratrol on monocyte adhesion was found to be concentration-dependent.

### 2.2. Resveratrol Suppressed Gene Expression of TNF-α-Induced Chemokine and Adhesion Molecules in ECs

**Figure 2:**
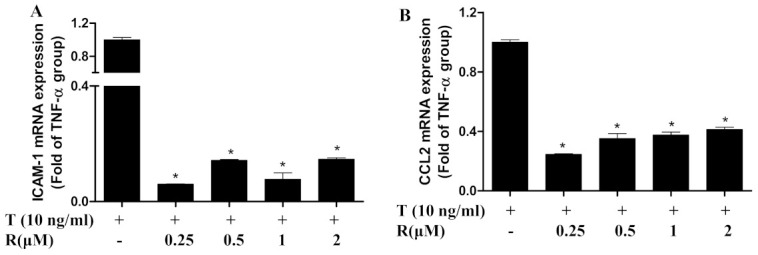
Resveratrol reduced the expression of ICAM-1 (**A**) and CCL2 (**B**) in ECs. EA.hy926 cells were pretreated with various concentrations of resveratrol (R) for 1 h prior to the addition of TNF-α (T, 10 ng/mL) for 1 h in the continued presence or absence of resveratrol. The relative mRNA abundances of ICAM-1 and CCL2 were determined by real-time PCR and mean quantities were normalized based on the mean of housekeeping gene GAPDH. T, TNF-α; R, Resveratrol. Values represent mean ± SEM, *n* = 3. *, *p* < 0.05 vs. control; *, *p* < 0.05 vs. TNF-α-alone-treated cells. CCL2/MCP-1, monocyte chemoattractant protein-1; ICAM-1, soluble intercellular adhesion molecule-1.

Prior to monocyte adhesion, monocytes are recruited to ECs through chemokines and adhesion molecules [35,36]. Real-time PCR determined that exposure of ECs to TNF-α for 1 h significantly increased mRNA expression of monocyte chemoattractant protein CCL2 and intercellular adhesion molecule ICAM-1 (Figure 2A,B). Pretreatment of resveratrol in concentrations as low as 0.25 μm markedly suppressed TNF-α-induced expression of these chemokines and adhesion molecules. These results indicate that pretreatment of resveratrol has an anti-inflammatory effect. 

### 2.3. Resveratrol Inhibits TNF-α-Induced NF-κB Activation in HUVECs

**Figure 3:**
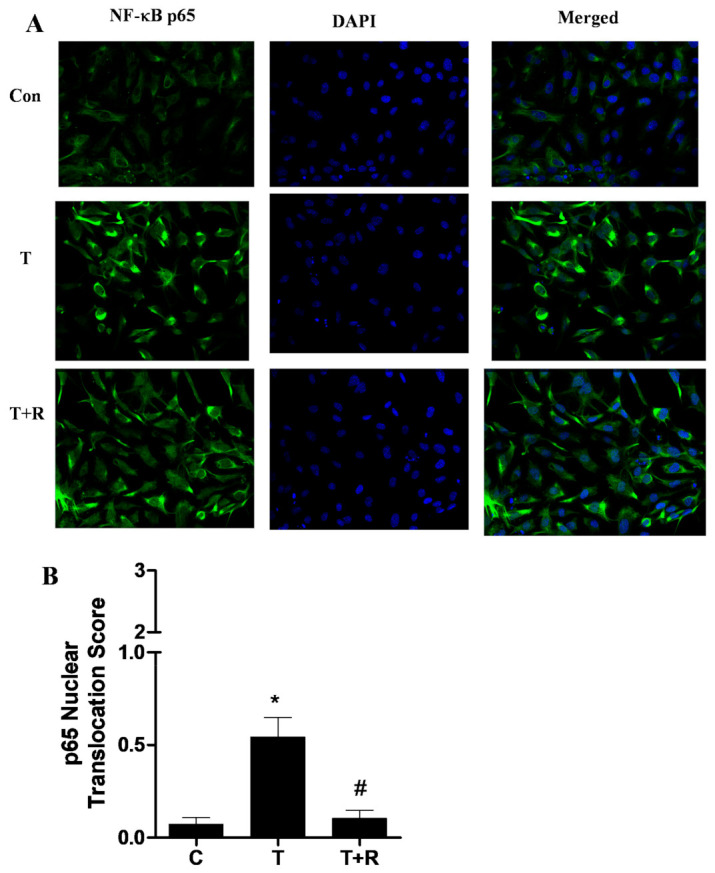
Resveratrol inhibited TNF-α-induced NF-κB signaling in HUVECs. (**A**) The cells were pretreated with 1 μm of resveratrol (R) for 1 h prior to the addition of TNF-α (T, 10 ng/mL) for 15 min in the continued presence or absence of resveratrol. Nuclear translocation of the NF-κB p65 subunit was visualized by immunofluorescence staining of ECs. Representative immunofluorescence fields show NF-κB p65 (green), nucleic acid with DAPI (blue), and overlay. (**B**) The nuclear and cytoplasmic fractions of p65 were quantified using a scoring system as described in Materials and Methods. *, *p* < 0.05 vs. control; #, *p* < 0.05 vs. TNF-α-alone-treated group.

NF-κB activation through nuclear translocation of the p65 heterodimer is an essential step in TNF-α-induced transcription of chemokines and adhesion molecules [23,24,25]. Thus, we investigated the role of resveratrol on TNF-α-stimulated activation of NF-κB signaling. Immunofluorescence-stained images of NF-κB p65 nuclear translocation showed that cells pretreated with resveratrol showed a significant reduction in positive fluorescence as compared to cells treated only with TNF-α (Figure 3A,B). These results suggest that resveratrol has a potent anti-inflammatory effect that is partly mediated through inhibition of the NF-κB signaling pathway.

### 2.4. Dietary Ingestion of Resveratrol Suppresses TNF-α-Induced Vascular Inflammation In Vivo

**Figure 4:**
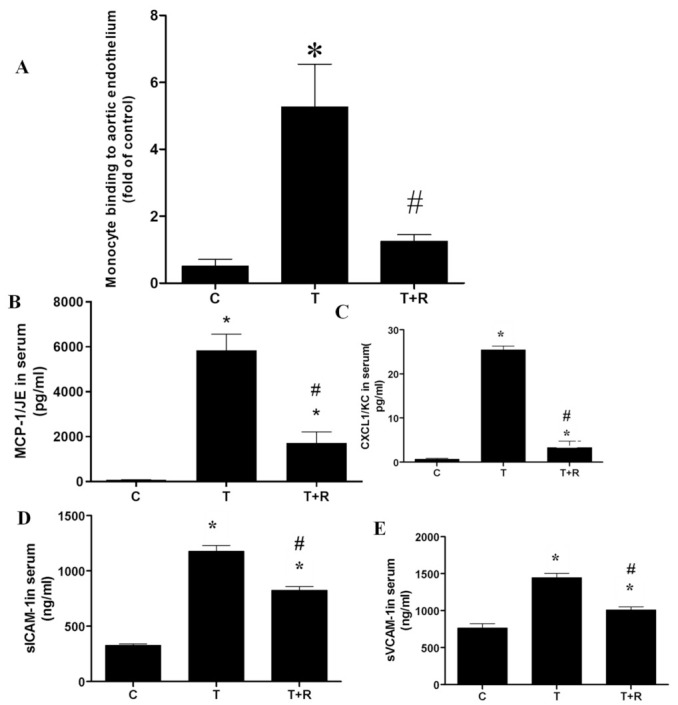
Dietary intake of resveratrol decreased monocyte binding to aortic endothelia (**A**), secretion of chemokines (**B**,**C**) and adhesion molecules (**D**,**E**) in the serum of TNF-α-treated mice. MCP-1/JE, CXCL1/KC, sICAM-1, and sVCAM-1 in serum were analyzed using ELISA. Values represent mean ± SEM. *, *p* < 0.05 vs. control; #, *p* < 0.05 vs. TNF-α-alone-treated mice. T, tumor necrosis factor-α; MCP-1/JE, mouse monocyte chemotactic protein 1/JE; CXCL1/KC, chemokine (C-X-C motif) ligand 1; sICAM-1, soluble intercellular adhesion molecule-1; sVCAM-1, soluble vascular adhesion molecule-1.

We further examined whether resveratrol could affect TNF-induced vascular inflammation in C57BL/6 mice. First, monocyte binding to mouse aortic endothelia Ex Vivo was evaluated using WEHI 78/24 monocytic cells. TNF-α treatment caused significantly increased monocyte adhesion to the endothelia of aortic cross-sections, which was largely reduced in mice fed 0.4% resveratrol in the diet (Figure 4A–E).

Previous studies indicated that chemokine MCP-1 and CXCL1/KC play a key role in monocyte recruitment, while adhesion molecules ICAM-1 and VCAM-1 are involved in ensuring firm adhesion of monocytes to the endothelial layer and subsequent transmigration into the intima of the artery [37,38]. As seen in Figure 4B–D, chemokines MCP-1/JE and CXCL1/KC (mouse homologs of human chemokine MCP-1) and adhesion molecules sICAM-1 and sVCAM-1 were present in significantly higher concentration in mice treated with TNF-α compared to that of the control group. However, in mice that were fed dietary supplementations of resveratrol, serum concentrations of MCP-1/JE, CXCL1/KC, sICAM-1 and sVCAM-1 regressed. Based on these results, we demonstrate that resveratrol attenuates endothelial inflammation partly by reducing chemokine and adhesion molecule production. 

**Figure 5:**
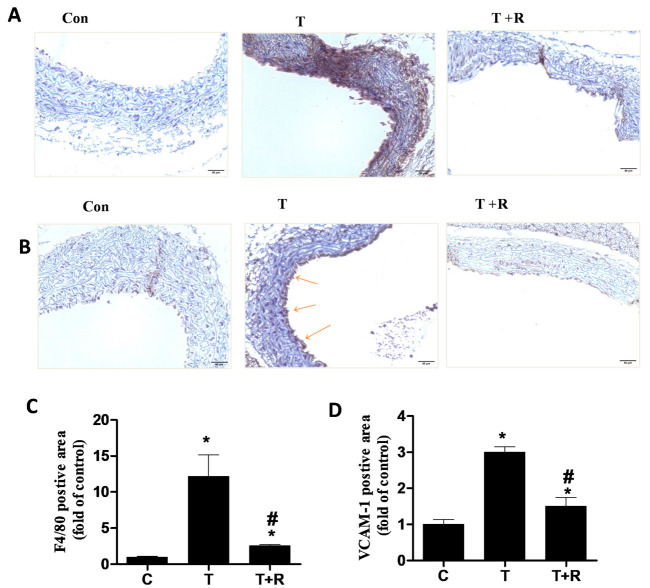
Representative images showing the immunohistochemical staining for F4/80-positive monocyte-derived macrophages (**A**) and adhesion molecule VCAM-1 (**B**) in aortic cross-sections of C57BL/6 mice. C57BL/6 mice were fed AIN-93G rodent diets with and without 0.4% resveratrol for one week followed by 25 μg/kg/day of TNF-α injected intraperitoneally for 7 days. After the treatment periods, the animals’ aortas were harvested for sectioning. Quantitative analysis of F4/80- (**C**) and VCAM-1- (**D**) positive areas were performed. Arrows indicate typical positive-stained regions at a magnification of 40× (scale bar = 50 μm). T, TNF-α; R, resveratrol; T + R, TNF-α + resveratrol, * *p* < 0.05 vs. control; #, *p* < 0.05 vs. TNF-α-alone-treated mice.

During inflammation, monocytes undergo sub-endothelial transmigration and differentiate into macrophages [35,36,37,38,39]. F4/80 is one of the most commonly used monocyte-derived macrophage markers [40]. To further corroborate the hypothesis that resveratrol suppresses inflammation In Vivo, immunohistochemistry was employed to assess the expression of vascular adhesion molecule VCAM-1 and monocyte-derived macrophage marker F4/80 in mouse aortic cross-sections (Figure 5A–D). As shown in Figure 5A–D, the aorta of mice administered with TNF-α displayed a high intensity of positive F4/80 and VCAM-1 staining, indicating high recruitment of monocytes to the aortic vessel and differentiation into macrophages. However, dietary supplementation of resveratrol significantly reduced the intensity of both F4/80 and VCAM-1 staining, confirming the anti-inflammatory properties of resveratrol (Figure 5A–D).

### 2.5. Resveratrol Prevents TNF-α-Induced Disruption of Aortic Elastin Fiber in Mouse Aortic Cross-Sections

**Figure 6:**
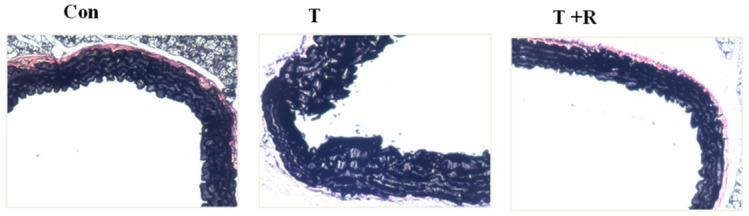
Representative aortic elastin fibers were visualized using Verhoeff–Van Gieson staining (magnification of 40×, scale bar = 50 μm). C57BL/6 mice were fed AIN-93G rodent diets with and without 0.4% resveratrol for one week followed by 25 μg/kg/day of TNF-α injected intraperitoneally for 7 days. After the treatment periods, the animals’ aortas were harvested for sectioning. T, TNF-α; R, resveratrol; T + R, TNF-α + resveratrol.

Histopathological examination of aortas using Verhoeff–Van Gieson staining revealed severe vascular structural abnormalities, primarily disruption and discontinuity of elastin fibers, in mice treated with TNF-α (Figure 6). Dietary ingestion of resveratrol significantly inhibited these structural abnormalities in the aortas and aided in the maintenance of the delicate organization of elastin fibers, comparable to that of the control group (Figure 6).

### 2.6. Resveratrol Diminishes TNF-α-Induced NF-κB Activation in Aortic Cross-Sections

**Figure 7:**
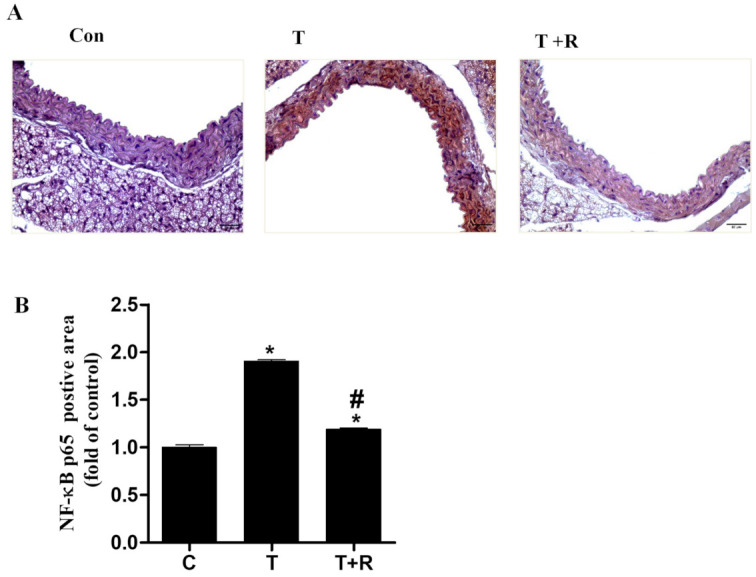
Representative images showing the immunohistochemical staining for NF-κB p65 in aortic cross-sections (magnification of 40×, scale bar = 50 μm). C57BL/6 mice were fed AIN-93G rodent diets with and without 0.4% resveratrol for one week followed by 25 μg/kg/day of TNF-α injected intraperitoneally for 7 days. After treatment periods, the animals’ aortas were harvested for sectioning. Representative photomicrographs of immunohistochemical staining for NF-κB p65 (**A**). Quantitative analysis of NF-κB p65 (**B**). T, TNF-α; T+R, TNF-α + resveratrol. *, *p* < 0.05 vs. control; #, *p* < 0.05 vs. TNF-α-alone-treated mice.

Immunohistochemistry was used to identify activation of the NF-κB p65 heterodimer in mice aortas. As displayed in Figure 7A,B, a strong NF-κB staining was present in the mouse aortic cross sections in the TNF-α-only treatment group, indicating inflammation in the aortic vessels. However, dietary supplementation of resveratrol significantly diminished the intensity of the staining, suggesting the inhibitory role of resveratrol in NF-κB signaling In Vivo.

## 3. Discussion

Extensive studies demonstrated that resveratrol, primarily consumed through grapes and red wine, exerts cardioprotective effects through its antioxidant and anti-inflammatory properties [23,24,25,26,27]. Its first recognized importance was in the early 1990s with the introduction of the “French paradox”, an incidence where the French population, despite having a regular high-fat diet, had a lower susceptibility to heart disease partially due to regular consumption of resveratrol-containing red wine [41]. However, the protective role of resveratrol against vascular inflammation and the underlying mechanism at physiologically achievable concentrations in a TNF-α-induced inflammatory model remains largely unknown to the best of our knowledge.

Numerous clinical studies reported low bioavailability of resveratrol in the body due to the rapid metabolism of trans-resveratrol into glucuronide and sulfate conjugates [31,42,43,44]. However, many previous studies showed the anti-inflammatory action of resveratrol in ECs using concentrations that are far beyond physiologically achievable through dietary intake [45,46]. In this research, we demonstrated that resveratrol at physiologically achievable concentrations (<5 μm) attenuates TNF-α-stimulated monocyte-EC adhesion. Clinical studies revealed that free and conjugated forms of resveratrol were present in plasma and urine samples of human subjects who orally consumed resveratrol [32,33], but only up to 2.4 μm of resveratrol was found in the plasma of human participants who orally ingested a high dose of resveratrol (5 g) [33]. Adhesion molecules such as VCAM-1 and ICAM-1 and chemokines such as MCP-1/JE and CXCL1/KC are important modulators in monocyte recruitment, rolling, and adhesion to the vascular endothelium and play a fundamental role in the pathogenesis of atherosclerosis [35,36,47]. We report that resveratrol suppressed TNF-α-induced increases in adhesion molecules and chemokines in ECs. Additionally, resveratrol reduced TNF-α-stimulated activation of NF-κB by inhibiting IκB-α degradation and subsequently preventing nuclear localization of NF-κB p65 subunits in ECs, suggesting that resveratrol may exert its anti-inflammatory effect by interfering with the NF-κB signal transduction pathway. Mice fed a diet of 0.4% resveratrol showed suppressed serum concentrations of adhesion molecules and chemokines as well as attenuated expression of VCAM-1- and F4/80-positive macrophages in the vascular tissue of aortic cross sections. Overall, our results suggest that resveratrol can be an easily attainable naturally occurring and low-cost compound that can be used to ameliorate atherosclerosis.

Endothelial dysfunction and monocyte recruitment is essential in the initiation and exacerbation of atherosclerosis [48]. Previous studies implied that up-regulated expression of adhesion molecules and chemokines are involved in endothelial dysfunction and chronic endothelial inflammation, hence aiding in the development of atherosclerosis and other cardiovascular diseases [8,49,50]. Adhesion molecules such as ICAM-1 and VCAM-1 are mediators that aid monocytes’ transition from rolling to firm arrest and subsequent transmigration into inflamed tissue [35]. In fact, elevated expression of these leukocyte adhesion molecules was reported in vascular-lesion-prone sites and in human coronary atherosclerotic plaques [51,52]. Additionally, C-C and C-X-C chemokines such as MCP-1 and IL-8 play a vital role in monocyte recruitment, rolling, and adhesion to vascular endothelial monolayers [35,37,47]. Here, we demonstrated that resveratrol significantly reduced TNF-α-activated mRNA expression of ICAM-1 and MCP-1 in ECs, suggesting that the anti-inflammatory effect of resveratrol may be partially due to a reduced production of proinflammatory adhesion molecules and chemokines. The in vitro results were recapitulated in the animal study, which showed that the up-regulated serum levels of MCP-1/JE, CXC1/KC, sVCAM-1, and sICAM-1 after TNF-α treatment was vastly attenuated in mice that were fed resveratrol. Murine animals do not have IL-8 but chemokine CXCL1/KC can act as a functional homolog [53]. These results suggest that resveratrol may be exerting its cardioprotective effects against vascular inflammation partially by preventing production and/or secretion of chemokines and adhesion molecules. Since adhesion molecules and chemokines are secreted by various cell types, these results alone are insufficient to pinpoint ECs as the target of resveratrol’s anti-inflammatory effects [54,55].

NF-κB is well-recognized as a key regulator of inflammation and has been implicated to be essential for the pathogenesis of atherosclerosis [56,57]. One of the ways in which NF-κB exerts its proatherogenic effects is by up-regulating the transcription of adhesion molecules (e.g., VCAM-1 and ICAM-1) and chemokines (e.g., MCP-1) [23]. As mentioned previously, these proinflammatory adhesion molecules and chemokines are critical in inducing inflammation due to their involvement in monocyte attraction and adhesion to the endothelium [35,36,47]. In fact, a previous In Vivo study reported that inhibition of NF-κB activation in ECs reduced formation of atherosclerotic plaques in atherosclerosis-prone mouse models [57]. Activation of the NF-κB pathway is mediated by diverse extracellular stimuli, including cytokines such as TNF-α [19,58]. In a normal unstimulated state, NF-κB is kept inactive in the cytoplasm by being bound to inhibitors such as IκB-α [23]. In the classical (canonical) pathway, the stimuli induce a signal transduction pathway that activates NF-κB by IKK complex-mediated phosphorylation and degradation of IκB-α [59]. As a result, NF-κB p50/65 heterodimers translocate into the nucleus, where they bind to promoters of NF-κB-induced proinflammatory genes such as MCP-1, TNF-α, and IL-6 [60]. Our immunofluorescence staining results suggested that resveratrol inhibited TNF-α-induced NF-κB 65 nuclear translocation in ECs. To the best of our knowledge, this is the first time that resveratrol at physiologically achievable concentrations is shown to prevent IκB-α degradation and subsequent NF-κB translocation into the nucleus in ECs. Immunohistochemical analyses of mouse aorta cross-sections additionally confirmed resveratrol’s inhibitory effect on NF-κB activation In Vivo. The aortic cross-sections of mice treated with TNF-α showed the high intensity of NF-κB p65 staining, indicative of TNF-α-induced inflammation in the aortic vascular wall. Mice given dietary resveratrol showed greatly reduced expression of NF-κB p65 staining compared to the TNF-α-only treatment group, suggesting that resveratrol may exert its anti-inflammatory properties by targeting NF-κB signaling, in line with in vitro results we have previously discussed above. However, the exact mechanism of how resveratrol interferes with the canonical NF-κB pathway is still unclear.

F4/80 is a well-characterized murine macrophage marker. In previous studies, F4/80-positive macrophages were present in elevated levels in highly inflamed mouse aortas, suggesting it may be a potential inflammatory marker [61,62]. Our immunohistochemical examination showed high abundance of F4/80-positive monocyte-derived macrophages and elevated VCAM-1 staining in mice aortic cross-sections when treated with TNF-α, suggesting that TNF-α treatment induced inflammation in aortic walls. However, mice that were fed dietary resveratrol showed a significant decrease in both F4/80-positive macrophages and VCAM-1 staining, suggesting that resveratrol may target the vascular wall to exert its protective effects against inflammation. Based on hematoxylin and eosin stains, TNF-α triggered extensive structural changes in the intima layer of the artery, implying endothelial injury. However, resveratrol supplementation significantly improved such structural damages. Verhoeff–Van Gieson staining revealed resveratrol’s ability to restore disruption of aortic elastin fiber in mice aortas induced by TNF-α. Although the exact mechanism is unknown, this restorative property may be linked in part to the ability of resveratrol in down-regulating cytokines and adhesion molecules and inhibiting the NF-κB signal pathway as discussed above.

In summary, this study demonstrates for the first time that dietary ingestion of resveratrol reduces vascular endothelial inflammation in mouse models by inhibiting NF-κB activation and reducing VCAM-1 and F4/80 expression in aortic tissue after TNF-α stimulation. Resveratrol at concentrations as low as 1 μm significantly suppressed TNF-α-induced EC-monocyte adhesion and endothelial expression of chemokines and adhesion molecules. We suggest a possible link between the ability of resveratrol to protect against vascular inflammation and its down-regulating effect of the NF-κB signaling pathway, but further studies are required to determine the exact mechanism. Our findings shed light on resveratrol as a potential novel therapeutic agent that can provide protection against inflammation and inflammatory diseases.

## 4. Materials and Methods

### 4.1. Chemicals and Materials

Dulbecco’s modified Eagle’s medium (DMEM), Calcein-AM (Calcein O, O’-diacetate tetrakis (acetoxymethyl) ester, and RPMI-1640 were purchased from Life Technologies (Grand Island, NY, USA). Enzyme-linked immunosorbent assay (ELISA) kits for human and mouse adhesion molecules ICAM-1 (sICAM-1) and VCAM-1 (sVCAM-1) and mouse chemokines MCP-1/JE and CXCL1/KC were obtained from R&D Systems (Minneapolis, MN, USA). Goat antirabbit IgG, DyLight™ 488 conjugated secondary antibody and goat antirabbit horseradish peroxidase (HRP) IgG secondary antibody were purchased from Thermo Fisher Scientific Inc. (Waltham, MA, USA). Primary antibodies for immunohistochemistry were acquired from Cell Signaling Technology, Inc. for NF-κB p65 and IκB-α (Danvers, MA, USA), from Santa Cruz Biotechnology for VCAM-1 (Santa Cruz, CA, USA), and from BMA Biomedicals for F4/80 (Augst, Switzerland). Resveratrol (≥98%, HPLC) was from the Stanford Chemicals Company (Irvine, CA, USA), and other general chemicals, including DAPI, were procured from Sigma-Aldrich (St. Louis, MO, USA)

### 4.2. Cell Culture

EA.hy926 cells (passage 3–5) were cultured in DMEM containing 100 ug/mL streptomycin, 100 U/mL penicillin, and 10% fetal bovine serum (FBS) in a humidified incubator at 37 °C in a 95% air/5% CO2 environment. Primary human umbilical vein endothelial cells (HUVECs, (passage 3–5)) were grown in M199 medium supplemented with endothelial growth supplement EGM2 and 2% FBS. WEHI 78/24 cells were provided by Dr. Judith A. Berliner from UCLA and were cultured in DMEM supplemented with 10% FBS. Lastly, THP-1 cells (passage 3–5) were grown in RPMI-1640 medium with 10% FBS.

### 4.3. Monocyte Adhesion Assay

Monocyte adhesion to ECs was determined by using THP-1 cells as previously described [63]. EA.hy926 cells were grown to confluence in 98-well plates and treated with various concentrations of resveratrol (1 μm, 5 μm, and 10 μm) for 1 h before the addition of 10 ng/mL of TNF-α. Cells were then incubated in medium containing TNF-α in the continued presence or absence of resveratrol for 24 h. EA.hy926 cells were gently washed with serum-free medium and then incubated with calcein-AM-labeled THP-1 cells (1 × 106/mL RPMI1640 medium containing 1% FBS) for 1 h. In order to discard unbound monocytes, the EC monolayer was gently washed with medium. Monocyte adhesion was determined through fluorescence measured using a BioTek Synergy 2 Multi-Mode Microplate Reader (Winooski, VT, USA) at excitation and emission wavelengths of 496 nm and 520 nm.

### 4.4. Reverse Transcription and RT-PCR

EA.hy926 endothelial cells were pretreated with various concentrations of resveratrol (0.25 μm, 0.5 μm, 1 μm, and 2 μm) for 1 h. Ten ng/mL of TNF-α was then added in the continued presence or absence of resveratrol for 1 h. Trizol reagent was used to extract total RNA per the manufacturer’s protocol. Complementary DNA was generated by reverse transcription using 1 μg of total RNA. Each well contained a reaction mixture of 10 μL of SYBR green, 5 μL of distilled autoclaved H2O, and 2 μL each of forward and reverse oligonucleotide primers. SYRBR Green RT-PCR Master Mix was used (Life Technologies, Grand Island, NY, USA). The primers used were ICAM-1 (forward, 5′-CTCCCTCTCGGGTCTCTCTC-3′;reverse,5′-ACT GTG GGG TTC AAC CTC TG-3′) and MCP-1 (forward, 5′-CCC CAG TCA CCT GCT GTT AT-3′; reverse, 5′-TGG AAT CCT GAA CCC ACTTC-3′). The amplification profile was 50 °C for 2 min, then 95 °C for 10 min, followed by 40 cycles of 94 °C for 15 s and 60 °C for 1 min. The average amounts of each chemokine and adhesion molecule were averaged then normalized to that of the control housekeeping gene GAPDH.

### 4.5. Confocal Immunofluorescence Study of NF-κB p65 Nuclear Translocation

HUVECs were pretreated with 1 μm of resveratrol for 1 h on eight-well chamber slides. The cells were then incubated with 10 ng/mL of TNF-α for 15 min in the continued presence or absence of resveratrol. Cells were washed with PBS then fixed with 100% ice-cold methanol. Blocking was carried out at room temperature for 30 min using 10% normal goat serum (Sigma, St. Louis, MO, USA). Rabbit anti-NF-κB p65 primary antibody was added and incubation occurred for 2 h at 4 °C. After three consecutive PBS washes, cells were incubated for 1 h with goat antirabbit IgG DyLight™ 488 conjugated secondary antibody. After the last wash with PBS, the chamber slides were mounted with Fluroshield with DAPI mounting medium (Sigma Chemicals, St. Louis, MO, USA), and NF-κB p65 was visualized using an Olympus Fluoview FV5OO/IX81 confocal microscope (Waltham, MA, USA). The localization of the p65 signal with respect to the nucleus was evaluated to score as follows: cytoplasm only (score 0); evenly appear in cytoplasm and nucleus (score 1); most appear in nucleus with mild cytoplasm (score 2); nucleus only (score 3). DAPI was used to determine the approximate location of the nucleus. Score were averaged and compared with control group.

### 4.6. Animal and Experimental Design

Male C57BL/6 mice (age 10 weeks) purchased from the Jackson Laboratory were housed in microisolator cages located in a pathogen-free animal facility. All animal procedures were approved by the Institutional Animal Care and Use Committee and performed in accordance with the National Institutes of Health Guidelines for the Care and Use of Laboratory Animals. The mice were randomly separated into three groups (control, TNF-α, TNF-α + resveratrol), with 6–8 mice per group. Mice were fed an ANI-93G rodent diet or basal-modified AIN-93G rodent diet containing 0.4% resveratrol (Dyet, Inc., Bethlehem, PA, USA) depending on their allocated group. The use of resveratrol dosage was based on previous publications [31,32,33,34,64,65,66,67,68]. After one week, the mice were injected intraperitoneally (i.p.) with 25 μg/kg/day of TNF-α (PeproTech Inc., Rocky Hill, NJ, USA) for 7 consecutive days. Previous studies have indicated that this dosage of TNF-α resulted in markedly elevated expression of intracellular adhesion molecules and vascular barrier dysfunction [69,70]. Control mice were injected i.p. with PBS for the same period of time. Throughout the i.p. administration process, mice were continually fed with either the control or resveratrol diet. For the entire duration of the study, body weight and feed intake were recorded weekly. Two hours after the last i.p. injection, all the mice were euthanized, and blood samples were collected. The serum was frozen at −80 °C for ELISA analysis.

### 4.7. Ex Vivo Monocyte Adhesion Assay

Aortas were isolated from euthanized mice. The surrounding connective tissue and fat were removed, and then the aorta was gently washed with ice-cold PBS twice. After being placed in DMEM at 37 °C for 10 min, the aorta was opened longitudinally and fixed with needles onto 4% agar in 35 mm plates. The aortic strip was placed in 1 mL of DMEM containing 1% heat-inactivated FBS. WEHI 78/24 monocytes were fluorescently labeled with calcein-AM by following the manufacturer’s instructions. Fluorescence-labeled WEHI monocytes (1 × 10^6^) were added to the aortic strip and incubated for 30 min. Non-adherent cells were washed away and the number of bound monocytes were examined using a confocal microscope. Data was quantified using the Image J software (Version 1.48k, 2013, National Institute of Mental Health, Bethesda, MD, USA).

### 4.8. Measurements of Chemokines and Adhesion Molecules

Serum concentrations of adhesion molecules (sVCAM-1 and sICAM-1) and chemokines (MCP-1/JE and CXCL1/KC) were detected using Quantikine ELISA Kits (R&D Systems, Minneapolis, MN, USA) and procedures were performed as per the manufacturer’s instructions. To determine serum concentrations, samples were plotted against standard curves.

### 4.9. Histology

The thoracic aorta was isolated, and adherent fat was removed. The aorta was incubated in 10% buffered formalin solution overnight. After overnight fixation, 5 μm of the proximal artery was sliced off and placed in 200-proof ethyl alcohol for 24 h. The sectioned aorta was embedded in paraffin and then stained with Verhoeff–Van Gieson stain for elastin and hematoxylin-eosin. Staining was performed at AML Labs (Baltimore, MD, USA) while following standard protocol. Aortic sections were visualized under a bright field EVOS XL microscope (AMG, Bothell, WA, USA).

### 4.10. Analysis pf VCAM-1, F4/80, and NF-κB p65 in Mice Aortas

Paraffin-embedded tissue sections of 5 μm were deparaffinized in xylene and rehydrated through graded concentrations of ethanol washes. Sections were then boiled in 10 mM sodium citrate buffer (pH 6.0) followed by cooling at room temperature for 30 min. The tissue sections were incubated in 3% H_2_O_2_ for 10 min and then placed in 5% normal goat serum (Vector Laboratories) in TBST for additional 30 min. Following these procedures, the tissue sections were incubated in primary antibodies overnight at 4 °C. For VCAM-1, rabbit anti-VCAM-1 primary antibody (1:1000 dilution, Santa Cruz Biotechnology) and the Vectastain Elite Rabbit IgG kit (Vector Laboratories) were used. For F4-80, a rat monoclonal anti-F4/80 primary antibody (1:50 dilution, Bachem) and the Vectastain Elite Rat IgG kit (Vector Laboratories) were used. For NF-κB p65, a rabbit monoclonal anti-NF-κB p65 primary antibody (1:50 dilution, Santa Cruz) and the Vectastain Elite rabbit IgG kit (Vector Laboratories) were used. Afterward, tissue sections were incubated in corresponding secondary antibodies from the rabbit/rat Vectastain ABC-AP kit (Vector Laboratories). Immunohistochemistry was visualized using 3,3′-diaminobenzidine (Dako) and Harris hematoxylin was used to counterstain the nuclei. Photomicrographs of stained mouse aortas were captured using an AMG EVOS XL digital inverted bright field and phase-contrast microscope (Bothell, WA, USA). Quantitative analysis of VCAM-1- and F4/80-positive areas in the aortas was carried out using the Image J software.

### 4.11. Statistical Analysis

All data are expressed as mean ± SEM. Statistical analyses were performed using ANOVA and the GraphPad Prism^®^ software (La Jolla, CA, USA). Significant treatment differences were subjected to Tukey’s multiple comparison tests. The level of statistical significance was set at *p* < 0.05.

## Data Availability

The data used to support the findings of this study are available from the corresponding author upon reasonable request.

## References

[1] Bonow R.O., Smaha L.A., Smith S.C., Mensah G.A., Lenfant C. (2002). World Heart Day 2002: The international burden of cardiovascular disease: Responding to the emerging global epidemic. Circulation.

[2] Tedgui A., Mallat Z. (2006). Cytokines in atherosclerosis: Pathogenic and regulatory pathways. Physiol. Rev..

[3] Virani S.S., Alonso A., Benjamin E.J., Bittencourt M.S., Callaway C.W., Carson A.P., Chamberlain A.M., Chang A.R., Cheng S., Delling F.N. (2020). Heart disease and stroke statistics—2020 update: A report from the American Heart Association. Circulation.

[4] Nordestgaard B.G., Zacho J. (2009). Lipids, atherosclerosis and CVD risk: Is CRP an innocent bystander?. Nutr. Metab. Cardiovasc. Dis..

[5] Frostegård J. (2013). Immunity, atherosclerosis and cardiovascular disease. BMC Med..

[6] Libby P., Ridker P.M., Maseri A. (2002). Inflammation and atherosclerosis. Circulation.

[7] Pearson T.A., Mensah G.A., Alexander R.W., Anderson J.L., Cannon R.O., Criqui M., Fadl Y.Y., Fortmann S.P., Hong Y., Myers G.L. (2003). Markers of inflammation and cardiovascular disease: Application to clinical and public health practice: A statement for healthcare professionals from the Centers for Disease Control and Prevention and the American Heart Association. Circulation.

[8] Steyers C.M., Miller F.J. (2014). Endothelial dysfunction in chronic inflammatory diseases. Int. J. Mol. Sci..

[9] Castellon X., Bogdanova V. (2016). Chronic inflammatory diseases and endothelial dysfunction. Aging Dis..

[10] Arida A., Protogerou A.D., Kitas G.D., Sfikakis P.P. (2018). Systemic inflammatory response and atherosclerosis: The paradigm of chronic inflammatory rheumatic diseases. Int. J. Mol. Sci..

[11] Cybulsky M.I., Iiyama K., Li H., Zhu S., Chen M., Iiyama M., Davis V., Gutierrez-Ramos J.-C., Connelly P.W., Milstone D.S. (2001). A major role for VCAM-1, but not ICAM-1, in early atherosclerosis. J. Clin. Investig..

[12] Picchi A., Gao X., Belmadani S., Potter B.J., Focardi M., Chilian W.M., Zhang C. (2006). Tumor necrosis factor-α induces endothelial dysfunction in the prediabetic metabolic syndrome. Circ. Res..

[13] Zhang H., Park Y., Wu J., Lee S., Yang J., Dellsperger K.C., Zhang C. (2009). Role of TNF-α in vascular dysfunction. Clin. Sci..

[14] Azzawi M., Hasleton P. (1999). Tumour necrosis factor alpha and the cardiovascular system: Its role in cardiac allograft rejection and heart disease. Cardiovasc. Res..

[15] Pande R.L., Brown J., Buck S., Redline W., Doyle J., Plutzky J., Creager M.A. (2015). Association of monocyte tumor necrosis factor α expression and serum inflammatory biomarkers with walking impairment in peripheral artery disease. J. Vasc. Surg..

[16] Ohta H., Wada H., Niwa T., Kirii H., Iwamoto N., Fujii H., Saito K., Sekikawa K., Seishima M. (2005). Disruption of tumor necrosis factor-α gene diminishes the development of atherosclerosis in ApoE-deficient mice. Atherosclerosis.

[17] Rastogi S., Rizwani W., Joshi B., Kunigal S., Chellappan S.P. (2012). TNF-α response of vascular endothelial and vascular smooth muscle cells involve differential utilization of ASK1 kinase and p73. Cell Death Differ..

[18] Barath P., Fishbein M.C., Cao J., Berenson J., Helfant R.H., Forrester J.S. (1990). Detection and localization of tumor necrosisfactor in human atheroma. Am. J. Cardiol..

[19] Liu T., Zhang L., Joo D., Sun S.-C. (2017). NF-κB signaling in inflammation. Signal Transduct. Target. Ther..

[20] Sasaki M., Ostanin D., Elrod J., Oshima T., Jordan P., Itoh M., Joh T., Minagar A., Alexander J. (2003). TNF-α-induced endothelial cell adhesion molecule expression is cytochrome P-450 monooxygenase dependent. Am. J. Physiol. Cell Physiol..

[21] Hayden M.S., Ghosh S. (2014). Regulation of NF-κB by TNF Family Cytokines. Semin. Immuno..

[22] Sakurada S., Kato T., Okamoto T. (1996). Induction of cytokines and ICAM-1 by proinflammatory cytokines in primary rheumatoid synovial fibroblasts and inhibition by N-acetyl-L-cysteine and aspirin. Int. Immunol..

[23] De Winther M.P., Kanters E., Kraal G., Hofker M.H. (2005). Nuclear factor κB signaling in atherogenesis. Arterioscler. Thromb. Vasc. Biol..

[24] Brand K., Page S., Rogler G., Bartsch A., Brandl R., Knuechel R., Page M., Kaltschmidt C., Baeuerle P.A., Neumeier D. (1996). Activated transcription factor nuclear factor-kappa B is present in the atherosclerotic lesion. J. Clin. Investig..

[25] Hajra L., Evans A.I., Chen M., Hyduk S.J., Collins T., Cybulsky M.I. (2000). The NF-κB signal transduction pathway in aortic endothelial cells is primed for activation in regions predisposed to atherosclerotic lesion formation. Proc. Natl. Acad. Sci. USA.

[26] Repossi G., Das U.N., Eynard A.R. (2020). Molecular Basis of the Beneficial Actions of Resveratrol. Arch. Med. Res..

[27] Cong X., Li Y., Lu N., Dai Y., Zhang H., Zhao X., Liu Y. (2014). Resveratrol attenuates the inflammatory reaction induced by ischemia/reperfusion in the rat heart. Mol. Med. Rep..

[28] Fan Y., Liu L., Fang K., Huang T., Wan L., Liu Y., Zhang S., Yan D., Li G., Gao Y. (2016). Resveratrol ameliorates cardiac hypertrophy by down-regulation of miR-155 through activation of breast cancer type 1 susceptibility protein. J. Am. Heart Assoc..

[29] Jia Z., Zhu H., Misra B.R., Mahaney J.E., Li Y., Misra H.P. (2008). EPR studies on the superoxide-scavenging capacity of the nutraceutical resveratrol. Mol. Cell. Biochem..

[30] Park D.-W., Baek K., Kim J.-R., Lee J.-J., Ryu S.-H., Chin B.-R., Baek S.-H. (2009). Resveratrol inhibits foam cell formation via NADPH oxidase 1-mediated reactive oxygen species and monocyte chemotactic protein-1. Exp. Mol. Med..

[31] Walle T., Hsieh F., DeLegge M.H., Oatis J.E., Walle U.K. (2004). High absorption but very low bioavailability of oral resveratrol in humans. Drug Metab. Dispos..

[32] Goldberg D.M., Yan J., Soleas G.J. (2003). Absorption of three wine-related polyphenols in three different matrices by healthy subjects. Clin. Biochem..

[33] Boocock D.J., Faust G.E., Patel K.R., Schinas A.M., Brown V.A., Ducharme M.P., Booth T.D., Crowell J.A., Perloff M., Gescher A.J. (2007). Phase I dose escalation pharmacokinetic study in healthy volunteers of resveratrol, a potential cancer chempreventive agent. Cancer Epidemiol. Prev. Biomark..

[34] Brown V.A., Patel K.R., Viskaduraki M., Crowell J.A., Perloff M., Booth T.D., Vasilinin G., Sen A., Schinas A.M., Piccirilli G. (2010). Repeat dose study of the cancer chemopreventive agent resveratrol in healthy volunteers: Safety, pharmacokinetics, and effect on the insulin-like growth factor axis. Cancer Res..

[35] Gerhardt T., Ley K. (2015). Monocyte trafficking across the vessel wall. Cardiovasc. Res..

[36] Mehra V.C., Ramgolam V.S., Bender J.R. (2005). Cytokines and cardiovascular disease. J. Leukoc. Biol..

[37] Gerszten R.E., Garcia-Zepeda E.A., Lim Y.-C., Yoshida M., Ding H.A., Gimbrone M.A., Luster A.D., Luscinskas F.W., Rosenzweig A. (1999). MCP-1 and IL-8 trigger firm adhesion of monocytes to vascular endothelium under flow conditions. Nature.

[38] Moss J.W., Ramji D.P. (2016). Cytokines: Roles in atherosclerosis disease progression and potential therapeutic targets. Future Med. Chem..

[39] Samson S., Mundkur L., Kakkar V.V. (2012). Immune response to lipoproteins in atherosclerosis. Cholesterol.

[40] Ma Y., Mouton A.J., Lindsey M.L. (2018). Cardiac macrophage biology in the steady-state heart, the aging heart, and following myocardial infarction. Transl. Res..

[41] Mukherjee S., Dudley J.I., Das D.K. (2010). Dose-dependency of resveratrol in providing health benefits. Dose-Response.

[42] Kuhnle G., Spencer J.P., Chowrimootoo G., Schroeter H., Debnam E.S., Srai S.K.S., Rice-Evans C., Hahn U. (2000). Resveratrol is absorbed in the small intestine as resveratrol glucuronide. Biochem. Biophys. Res. Commun..

[43] Miksits M., Maier-Salamon A., Aust S., Thalhammer T., Reznicek G., Kunert O., Haslinger E., Szekeres T., Jaeger W. (2005). Sulfation of resveratrol in human liver: Evidence of a major role for the sulfotransferases SULT1A1 and SULT1E1. Xenobiotica.

[44] Smoliga J.M., Blanchard O. (2014). Enhancing the delivery of resveratrol in humans: If low bioavailability is the problem, what is the solution?. Molecules.

[45] Chung E.Y., Kim B.H., Hong J.-T., Lee C.-K., Ahn B., Nam S.-Y., Han S.-B., Kim Y. (2011). Resveratrol down-regulates interferon-γ-inducible inflammatory genes in macrophages: Molecular mechanism via decreased STAT-1 activation. J. Nutr. Biochem..

[46] Liu C.-W., Sung H.-C., Lin S.-R., Wu C.-W., Lee C.-W., Lee I.-T., Yang Y.-F., Yu I.-S., Lin S.-W., Chiang M.-H. (2017). Resveratrol attenuates ICAM-1 expression and monocyte adhesiveness to TNF-α-treated endothelial cells: Evidence for an anti-inflammatory cascade mediated by the miR-221/222/AMPK/p38/NF-κB pathway. Sci. Rep..

[47] Zhang X.W., Liu Q., Wang Y., Thorlacius H. (2001). CXC chemokines, MIP-2 and KC, induce P-selectin-dependent neutrophil rolling and extravascular migration in vivo. Br. J. Pharmacol..

[48] Hadi H.A., Carr C.S., Al Suwaidi J. (2005). Endothelial dysfunction: Cardiovascular risk factors, therapy, and outcome. Vasc. Health Risk Manag..

[49] Krieglstein C.F., Granger D.N. (2001). Adhesion molecules and their role in vascular disease. Am. J. Hypertens..

[50] Yang X., Chang Y., Wei W. (2016). Endothelial dysfunction and inflammation: Immunity in rheumatoid arthritis. Mediat. Inflamm..

[51] O’Brien K.D., McDonald T.O., Chait A., Allen M.D., Alpers C.E. (1996). Neovascular expression of E-selectin, intercellular adhesion molecule-1, and vascular cell adhesion molecule-1 in human atherosclerosis and their relation to intimal leukocyte content. Circulation.

[52] Nakashima Y., Raines E.W., Plump A.S., Breslow J.L., Ross R. (1998). Upregulation of VCAM-1 and ICAM-1 at atherosclerosis-prone sites on the endothelium in the ApoE-deficient mouse. Arterioscler. Thromb. Vasc. Biol..

[53] Hol J., Wilhelmsen L., Haraldsen G. (2010). The murine IL-8 homologues KC, MIP-2, and LIX are found in endothelial cytoplasmic granules but not in Weibel-Palade bodies. J. Leukoc. Biol..

[54] Dustin M.L., Rothlein R., Bhan A.K., Dinarello C.A., Springer T.A. (1986). Induction by IL 1 and interferon-gamma: Tissue distribution, biochemistry, and function of a natural adherence molecule (ICAM-1). J. Immunol..

[55] Deshmane S.L., Kremlev S., Amini S., Sawaya B.E. (2009). Monocyte chemoattractant protein-1 (MCP-1): An overview. J. Interferon Cytokine Res..

[56] Yu X.-H., Zheng X.-L., Tang C.-K. (2015). Nuclear factor-κB activation as a pathological mechanism of lipid metabolism and ath-erosclerosis. Adv. Clin. Chem..

[57] Gareus R., Kotsaki E., Xanthoulea S., van der Made I., Gijbels M.J., Kardakaris R., Polykratis A., Kollias G., de Winther M.P., Pasparakis M. (2008). Endothelial cell-specific NF-κB inhibition protects mice from atherosclerosis. Cell Metab..

[58] Bouwmeester T., Bauch A., Ruffner H., Angrand P.-O., Bergamini G., Croughton K., Cruciat C., Eberhard D., Gagneur J., Ghidelli S. (2004). A physical and functional map of the human TNF-α/NF-κB signal transduction pathway. Nat. Cell Biol..

[59] Monaco C., Andreakos E., Kiriakidis S., Mauri C., Bicknell C., Foxwell B., Cheshire N., Paleolog E., Feldmann M. (2004). Canonical pathway of nuclear factor κB activation selectively regulates proinflammatory and prothrombotic responses in human atherosclerosis. Proc. Natl. Acad. Sci. USA.

[60] Li Y., Reddy M.A., Miao F., Shanmugam N., Yee J.-K., Hawkins D., Ren B., Natarajan R. (2008). Role of the histone H3 lysine 4 methyltransferase, SET7/9, in the regulation of NF-κB-dependent inflammatory genes relevance to diabetes and inflammation. J. Biol. Chem..

[61] Shaposhnik Z., Wang X., Lusis A.J. (2010). Arterial colony stimulating factor-1 influences atherosclerotic lesions by regulating monocyte migration and apoptosis. J. Lipid Res..

[62] Wu D., Nishimura N., Kuo V., Fiehn O., Shahbaz S., Van Winkle L., Matsumura F., Vogel C.F.A. (2011). Activation of aryl hydrocarbon receptor induces vascular inflammation and promotes atherosclerosis in apolipoprotein E−/− mice. Arterioscler. Thromb. Vasc. Biol..

[63] Jia Z., Babu P.V.A., Si H., Nallasamy P., Zhu H., Zhen W., Misra H.P., Li Y., Liu D. (2013). Genistein inhibits TNF-α-induced endothelial inflammation through the protein kinase pathway A and improves vascular inflammation in C57BL/6 mice. Int. J. Cardiol..

[64] Crowell J.A., Korytko P.J., Morrissey R.L., Booth T.D., Levine B.S. (2004). Resveratrol-associated renal toxicity. Toxicol. Sci..

[65] Howells L.M., Berry D.P., Elliott P.J., Jacobson E.W., Hoffmann E., Hegarty B., Brown K., Steward W., Gescher A.J. (2011). Phase I randomized, double-blind pilot study of micronized resveratrol (SRT501) in patients with hepatic metastases—Safety, pharmacokinetics, and pharmacodynamics. Cancer Prev. Res..

[66] Tauriainen E., Luostarinen M., Martonen E., Finckenberg P., Kovalainen M., Huotari A., Herzig K.H., Lecklin A., Mervaala E. (2011). Distinct effects of calorie restriction and resveratrol on diet-induced obesity and Fatty liver formation. J. Nutr. Metab..

[67] Ungvari Z., Bagi Z., Feher A., Recchia F.A., Sonntag W.E., Pearson K., de Cabo R., Csiszar A. (2010). Resveratrol confers endothelial protection via activation of the antioxidant transcription factor Nrf2. Am. J. Physiol. Heart Circ. Physiol..

[68] Abraham J., Johnson R.W. (2009). Consuming a diet supplemented with resveratrol reduced infection-related neuroinflammation and deficits in working memory in aged mice. Rejuvenation Res..

[69] Jia Z., Nallasamy P., Liu D., Shah H., Li J.Z., Chitrakar R., Si H., McCormick J., Zhu H., Zhen W. (2015). Luteolin protects against vascular inflammation in mice and TNF-alpha-induced monocyte adhesion to endothelial cells via suppressing IΚBα/NF-κB signaling pathway. J. Nutr. Biochem..

[70] Nallasamy P., Si H., Babu P.V.A., Pan D., Fu Y., Brooke E.A., Shah H., Zhen W., Zhu H., Liu D. (2014). Sulforaphane reduces vascular inflammation in mice and prevents TNF-α-induced monocyte adhesion to primary endothelial cells through interfering with the NF-κB pathway. J. Nutr. Biochem..

